# Early Outcomes of Cruciate-Retaining Versus Posterior-Stabilized Total Knee Arthroplasty in Younger Patients: A Prospective Eastern European Cohort Study

**DOI:** 10.3390/jcm14248893

**Published:** 2025-12-16

**Authors:** Lorand Vitalis, Andrei Marian Feier, Sandor György Zuh, Octav Marius Russu, Tudor Sorin Pop

**Affiliations:** 1 Doctoral School, George Emil Palade University of Medicine, Pharmacy, Science, and Technology of Targu Mures, 540142 Targu Mures, Romania; dr.vitalis.lorand@gmail.com; 2 Department of Orthopaedics and Traumatology, Clinical County Hospital of Mureș, 540139 Targu Mures, Romania; 3 Department M4 Clinical Sciences, Orthopedics and Traumatology I, George Emil Palade University of Medicine, Pharmacy, Science, and Technology of Targu Mures, 540139 Targu Mures, Romania; sandor.zuh@umfst.ro (S.G.Z.); octav.russu@umfst.ro (O.M.R.); tudor.pop@umfst.ro (T.S.P.)

**Keywords:** total knee arthroplasty, cruciate-retaining, posterior-stabilized, knee osteoarthritis, functional outcomes, younger patients

## Abstract

**Background/Objectives**: Cruciate-retaining (CR) and posterior-stabilized (PS) total knee arthroplasty (TKA) are both widely used in primary knee osteoarthritis (KOA), but evidence in younger patients remains limited. This study compared functional outcomes, pain, range of motion, quality of life, and psychological status between CR and PS implants in an Eastern European cohort. **Methods**: A prospective comparative cohort study was conducted in patients aged 40–64 years undergoing primary cemented TKA. The primary outcome was change in the Lower-Extremity Functional Scale (LEFS) at 12 months. Secondary outcomes included the Lysholm Knee Scoring Scale, EQ5D5L index, visual analogue scale (VAS) for pain, PROMIS Depression score, active knee flexion, and patient satisfaction. Outcomes were evaluated at baseline, 6 weeks, 3 months, 6 months, and 12 months. Between-group comparisons used Welch *t*-tests and results are reported as mean differences with 95% confidence intervals. **Results**: A total of 147 patients were included (CR *n* = 71; PS *n* = 76). The prespecified primary endpoint, 12-month change in LEFS, was very similar between groups (mean difference 0.14 points, 95% CI −3.80 to 4.08; *p* = 0.94). LEFS improved from 49.1 ± 14.8 to 66.8 ± 11.6 in the CR group and from 47.9 ± 14.6 to 65.8 ± 12.4 in the PS group at 12 months. Lysholm scores increased to 88.5 ± 11.4 (CR) and 86.2 ± 10.6 (PS) (*p* = 0.21). EQ-5D-5L improved in both groups, with a non-significant difference at 12 months (*p* = 0.077). VAS pain decreased from 7.39 ± 1.19 to 1.59 ± 0.84 (CR) and from 7.55 ± 1.46 to 1.75 ± 0.90 (PS) (*p* = 0.27). Active flexion increased to 117.5 ± 10.5° (CR) and 115.0 ± 11.3° (PS) (*p* = 0.15). PROMIS Depression improved similarly in both groups, and satisfaction levels at 12 months were comparable. **Conclusions**: Both CR and PS TKA produced comparable improvements in pain, function, quality of life, mental health, and knee flexion in KOA patients aged 40–64 at one year. Implant design did not influence clinical benefit or PROMs in this cohort.

## 1. Introduction

Knee osteoarthritis (KOA) in younger patients, generally defined as individuals under 65 years of age, poses significant clinical challenges [1]. These patients often experience reduced quality of life, higher psychological distress, and substantial work-related disability with socioeconomic consequences [1]. Although initial management typically involves nonpharmacological interventions, surgical options become increasingly relevant as disease severity progresses towards advanced stages [2,3]. When total knee arthroplasty (TKA) becomes necessary, younger recipients face higher rates of early revision, periprosthetic infection, and mechanical failure and report slightly lower satisfaction than older adults [4,5,6,7]. Posterior-stabilized (PS) and cruciate-retaining (CR) TKA designs are selected based on the ligament integrity and offer differing approaches to knee kinematics and load distribution [8]. By preserving the posterior cruciate ligament (PCL), CR TKA maintains more physiological femoral rollback and near-native tibiofemoral kinematics, which facilitate higher flexion angles [9]. Younger patients typically have greater activity levels and higher functional demands; therefore, even small advantages in proprioception and knee mechanics may translate into measurable differences in functional performance [10]. Existing comparative literature between PS and CR prostheses in younger, more active patients remains inconclusive, with limited outcome data specific to younger populations. Several studies report similar patient-reported outcomes (PROMs), return-to-sport rates, and implant survivorship between PS and CR designs in elderly populations. Although CR implants may offer greater postoperative flexion, differences in functional outcomes and complications appear clinically negligible [11,12]. Most comparative PS-CR studies focus on older adults, and younger patients usually form only small subgroups, limiting the ability to detect true differences. Very few studies have examined design choices specifically in patients under 65 years old, and those that have suggest outcomes are comparable in the short-to-mid term [4]. Given that TKA utilization among younger adults has surged and that younger patients face higher revision rates and activity expectations, the evidence gap is significant [5]. Comparative data on this cohort represent only a small fraction of what is available for older cohorts [6]. This imbalance supports a significant knowledge gap and the need for studies focusing on CR vs. PS TKA outcomes in the under 65 population. Given these existing uncertainties and limitations in current research regarding outcomes, patient satisfaction, functional recovery, and implant durability in younger, active OA populations, this study aimed to prospectively compare functional and clinical outcomes of PS versus CR knee prostheses in an Eastern European population. The primary objective was to evaluate between-group differences in PROMs over 12 months. Secondary objectives included knee range of motion, functional performance, stability, and satisfaction. We hypothesized that CR would yield modestly greater flexion and functional performance.

## 2. Materials and Methods

### 2.1. Ethics and Study Design

A STROBE-compliant prospective comparative cohort investigation was carried out in the Department of Orthopaedic and Trauma Surgery Clinical County Hospital of Mures from 15 April 2022 to 31 March 2025. This study was approved by the Clinical County Hospital of Mureș Ethical Committee (approval no. 5014/15.04.2022 and approval no. 9965/22.07.2025) and was conducted in accordance with the Declaration of Helsinki. All participants provided written informed consent at the time of enrollment.

### 2.2. Participants

**Figure 1:**
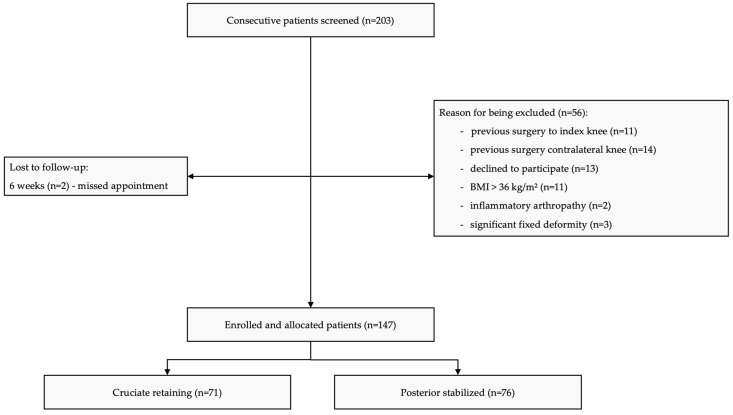
CONSORT diagram of patient screening, exclusion, and allocation for the study cohort.

All consecutive patients aged 40–64 years scheduled for a primary cemented TKA for Ahlbäck grade III, IV, or V primary knee OA were screened (Figure 1). This 40–64-year range was chosen a priori to include a younger, working age cohort, and exclude very young (<40 years) patients who often have different underlying etiologies and activity expectations. Candidates had to present with unilateral OA, intact neurovascular status, and the ability and willingness to comply with the full one-year follow-up.

Exclusion criteria were any inflammatory arthropathy, previous open surgery on the target or contralateral knee, extraarticular deformity demanding a constrained implant, active or past joint infection, neuromuscular or systemic conditions impairing gait, a body mass index > 36 kg/m^2^, fixed varus/valgus deformity > 20°, flexion contracture > 30°, sagittal range of motion < 60°.

### 2.3. Implant Selection

Implant design was determined intraoperatively according to a predefined algorithm and was not influenced by surgeon preference. Although all patients had advanced OA (Ahlbäck III–V), implant selection was based on direct intraoperative assessment of PCL integrity and gap balancing and not on OA grade alone. All enrolled patients fulfilled the same preoperative inclusion and exclusion criteria and were considered suitable for either a CR or PS design, with both implant types available for the planned surgery. A CR implant was chosen when the PCL appeared macroscopically intact and functional, when extension and flexion gaps were balanced after bone cuts, and when there was no major posterior femoral condylar bone loss. Preservation of a physiological posterior tibial slope and stable posterior translation and rollback during trial reduction were required before confirming the CR design. Conversely, a PS design was selected whenever the PCL was attenuated, frayed, hypertrophic or deficient, when residual laxity or instability persisted in flexion and/or extension despite appropriate bony resections, or when varus/valgus deformity (>10°), flexion contracture, or posterior condylar bone loss raised concern about reliable gap balancing. PS implants were also preferred if intraoperative trialing indicated the need for more predictable femoral rollback or if specific stability tests (including a positive pull-out–lift-off POLO test) suggested insufficient PCL function. Both surgeons used the same predefined intraoperative algorithm.

### 2.4. Surgical Procedure

**Figure 2:**
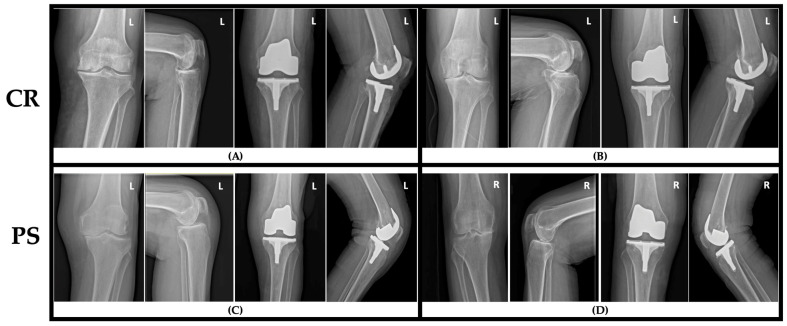
Panels (CR, **A**) and (CR, **B**) show two different patients treated with a cruciate-retaining implant, with representative anteroposterior and lateral radiographs obtained preoperatively and at 24 h postoperatively. Panels (PS, **C**) and (PS, **D**) show two different patients who underwent posterior-stabilized TKA with perioperative anteroposterior and lateral views. The images show the typical radiographic appearance and component positioning associated with CR and PS designs from Smith & Nephew Legion Genesis II implant system.

All procedures were performed by one of two high-volume arthroplasty consultants (S.Z.G, T.S.P, each > 100 TKAs/year), using a standard midline skin incision and medial parapatellar arthrotomy. Intramedullary femoral and extramedullary tibial guides targeted neutral mechanical alignment (180°) with a posterior tibial slope of 3°. Components were cemented with antibiotic loaded polymethylmethacrylate applied to both bone surfaces and implant undersides. Patellae were not resurfaced. The same implant system and polyethylene insert portfolio (Smith & Nephew, Watford, England, Legion Genesis II CR/PS) were used in both groups to minimize design-related bias (Figure 2).

Standard perioperative management included intravenous cefuroxime prophylaxis for 24 h, and tranexamic acid (2 × 1 g) and combined mechanical and pharmacological thromboprophylaxis for 30 days.

### 2.5. Rehabilitation

Rehabilitation was identical in both groups and initiated within 24 h postoperatively according to a standardized institutional protocol [13]. During the initial 7 day in-hospital phase, patients performed supervised active and assisted range of motion exercises, isometric quadriceps activation, and gait training with walking aids. Early full weight-bearing was encouraged as tolerated from day one. Continuous passive motion was used to achieve at least 90° of flexion by day 3. After discharge, patients followed a structured 12-week outpatient program focusing on progressive strengthening, balance training, and functional task practice. Discharge from hospital required independent transfers, ambulation over 30 m with aids, knee flexion over 90°, and a dry wound.

### 2.6. Outcomes

The prespecified primary outcome was the one-year change in the Lower-Extremity Functional Scale (LEFS). LEFS instrument contains twenty items scored from zero to four, producing a total score between 0 and 80 where higher values indicate better functional capacity. A change of approximately 9 points is accepted as the minimal clinically important difference (MCID) [14]. Additional evaluated outcomes included the Lysholm Knee Scoring Scale, EQ-5D-5L index, visual analogue scale (VAS) for pain, PROMIS Depression short form, active knee flexion and extension measured using a long-arm goniometer, patient satisfaction rated on a 0–10 scale, and perioperative complications. Baseline measurements were obtained within four weeks prior to surgery with identical assessments repeated at 6 weeks, and at 3, 6, and 12 months by research assistants (A.M.F, O.M.R) who remained blinded to the implant design throughout the study. All postoperative assessments were conducted on site at our institution. Follow-up adherence was ensured through a structured appointment confirmation system involving automated SMS reminders and direct phone calls. When a patient was unable to attend the scheduled visit, the appointment was rescheduled within a predefined follow-up window of seven days. Patients who missed a visit and could not return within the allowable window were excluded prior to analysis.

### 2.7. Statistical Analysis

Continuous variables are presented as mean ± standard deviation, and categorical variables as counts and percentages. Given the complete-case analysis design (excluding patients with missing longitudinal data), independent between-group (CR vs. PS) comparisons at each timepoint were performed using Welch’s *t*-test for continuous outcomes and chi-square or Fisher exact test for categorical outcomes. For secondary endpoints involving repeated assessments over time, we prioritized the evaluation of clinically distinct recovery milestones. To account for the longitudinal nature of the data and control for Type I errors without using complex model-based assumptions, multiplicity was controlled by applying the Holm–Bonferroni correction to the set of between-group comparisons at each timepoint. 95% CIs were calculated for mean differences. A *p* < 0.05 was considered statistically significant. Longitudinal results are reported descriptively at all scheduled timepoints. We prespecified the primary endpoint as the between-group difference in 12-month LEFS. Based on published metrics for LEFS, we set the MCID at 9 points and assumed an SD between 12 and15 points for KOA populations [14,15]. Using a two-sided alpha = 0.05 and 80% power for an unequal variance two-sample *t*-test, the required sample size was *n* = 28–44 per group. With our actual sample sizes, the minimum detectable difference at 80% power is ±5.6–6.9 points (SD = ±12–15), and at 90% power the requirement for a 9-point difference is ±59 per group. MCID proportions were presented with Wilson 95% CI, and between-group differences in MCID were analyzed using Newcombe–Wilson intervals. Changes in LEFS from baseline were compared between groups using Welch’s *t*-test with corresponding 95% CI.

## 3. Results

The two groups were well matched at baseline regarding age, BMI, sex distribution, smoking and alcohol consumption, rural versus urban residence, and employment status (Table 1).

The mean age was 57.2 ± 6.1 years in the PS group and 58.7 ± 6.4 years in the CR group, with a similar BMI profile. The proportion of female patients was slightly higher in both groups (53.9% vs. 60.6%) and no relevant differences were observed in lifestyle or socioeconomic factors.

OA severity was similar between groups. All patients had advanced Ahlbäck grade III-V KOA, and the distribution of grades did not differ significantly between the CR and PS cohorts (Table 2).

**Figure 3:**
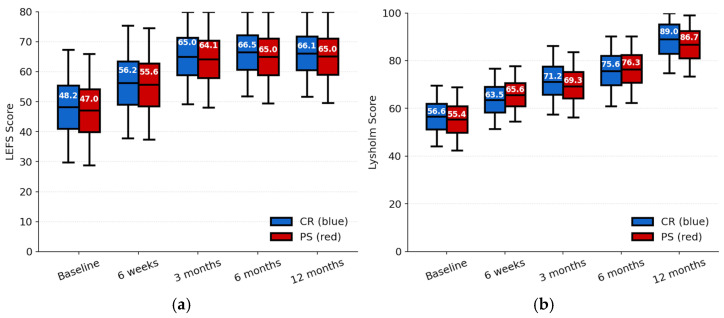
Functional outcome scores following TKA. (**a**) Lower-Extremity Functional Scale and (**b**) Lysholm Knee Scoring Scale over time. Values are presented as mean ± SD. CR (blue bars) and PS (red bars).

The primary outcome, 12-month change in the LEFS was similar between groups. Mean improvement was 17.74 ± 12.16 points in the CR cohort and 17.88 ± 12.28 points in the PS cohort. The one year between-group difference in change was 0.14 points (95% CI −3.80 to 4.08; *p* = 0.944, Cohen’s *d* = 0.01). In the CR group, mean LEFS increased from 48.20 ± 14.81 at baseline to 56.20 ± 14.84 at 6 weeks, 65.04 ± 12.83 at 3 months, 66.51 ± 11.84 at 6 months, and 66.14 ± 11.61 at 12 months (Figure 3a). In the PS group, LEFS increased from 47.09 ± 14.65 at baseline (*p* = 0.63) to 55.64 ± 14.65 at 6 weeks, 64.13 ± 12.90 at 3 months, 65.08 ± 12.46 at 6 months, and 65.02 ± 12.43 at 12 months. At all timepoints, between-group differences remained small (3 months: *p* = 0.67; 6 months: *p* = 0.47; 12 months: *p* = 0.60, −1.18 to −1.46 points, Cohen’s *d* = 0.08) and not statistically significant with all corresponding 95% CI crossing zero. Between-group differences in LEFS MCID improvement were minimal at all follow-ups (Table 3). Recovery favored PS slightly at 6 weeks (+0.57 points; *p* = 0.113), but differences at 3, 6, and 12 months were small and nonsignificant. MCID achievement rates were also similar between groups.

In the CR group, Lysholm scores increased from 56.61 ± 10.01 at baseline to 63.55 ± 10.04 at 6 weeks (*p* = 0.19 vs. PS), 71.20 ± 11.41 at 3 months (*p* = 0.30), 75.66 ± 11.21 at 6 months (*p* = 0.70), and 89.01 ± 11.44 at 12 months (*p* = 0.21) and in the PS group, scores increased from 55.41 ± 10.42 at baseline (*p* = 0.48 vs. CR) to 65.61 ± 9.12 at 6 weeks, 69.32 ± 10.81 at 3 months, 76.34 ± 10.71 at 6 months, and 86.71 ± 10.61 at 12 months (Figure 3b). All between-group mean differences (−1.2 to +2.1 points, Cohen’s *d* = 0.21) had 95% CI that included zero, indicating no evidence of superiority of either implant design in terms of PROMs.

**Figure 4:**
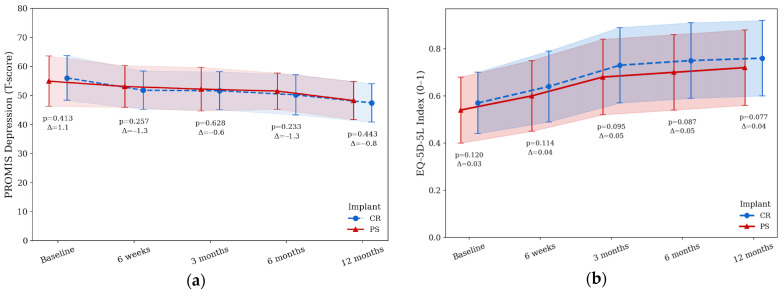
Patient reported quality of life and mental health outcomes after TKA in both groups. (**a**) PROMIS Depression T-scores over time for patients receiving posterior-stabilized (PS, triangles, solid line) or cruciate-retaining (CR, circles, dashed line) TKA; (**b**) EQ5D5L mean index over time. Data are presented as mean ± standard deviations. *p*-values indicate group comparisons at each follow-up timepoint.

PROMIS Depression scores decreased in both groups with an overall reduction in depressive symptoms (Figure 4a).

Mean baseline values were 56.1 ± 7.7 for the CR group and 55.0 ± 8.7 for the PS group, falling to 47.5 ± 6.6 and 48.3 ± 6.6 at 12 months. No significant differences between groups were observed at any follow-up point (Welch *t*-tests at each timepoint: all *p* > 0.2; Figure 4a). EQ-5D-5L health utility index improved over 12 months (CR: 0.57 ± 0.13 to 0.76 ± 0.16; PS: 0.54 ± 0.14 to 0.72 ± 0.16). Group means were slightly higher in the CR cohort at most timepoints; the between-group difference at 12 months did not reach significance (*p* = 0.077, Cohen’s *d* = 0.25; Welch *t*-test; Figure 4b).

**Figure 5:**
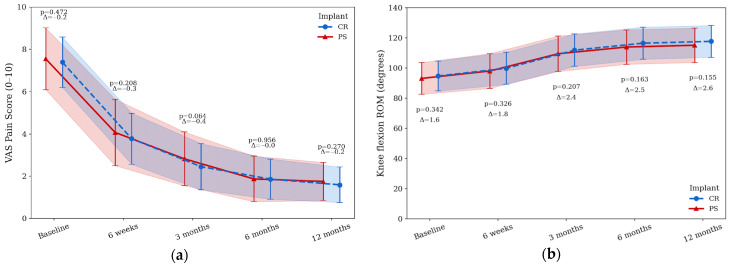
Pain reduction and functional mobility after TKA. (**a**) VAS pain scores and (**b**) knee flexion range of motion degrees over each timeline for PS (triangles, solid line) or CR (circles, dashed line). Data are presented as mean ± standard deviations. *p*-values indicate group comparisons at each follow-up timepoint.

VAS scores decreased in both groups over time. At baseline, VAS was 7.39 ± 1.19 in the CR group and 7.55 ± 1.46 in the PS group (*p* = 0.47) (Figure 5a). VAS reduced to 3.77 ± 1.21 vs. 4.07 ± 1.57 at 6 weeks (*p* = 0.20), 2.45 ± 1.09 vs. 2.82 ± 1.27 at 3 months (*p* = 0.06), 1.86 ± 0.95 vs. 1.87 ± 1.08 at 6 months (*p* = 0.95), and 1.59 ± 0.84 vs. 1.75 ± 0.90 at 12 months (*p* = 0.27) for CR and PS, respectively. No statistically significant between-group differences in pain were detected at final follow-up (*p* = 0.27, Cohen’s *d* = 0.18).

Active baseline flexion was 94.6 ± 9.9° in the CR group and 93.0 ± 10.5° in the PS group (*p* = 0.34) (Figure 5b). At 6 weeks, flexion reached 99.7 ± 10.5° vs. 97.9 ± 11.5° (*p* = 0.32); at 3 months, 111.7 ± 10.7° vs. 109.3 ± 11.7° (*p* = 0.20); at 6 months, 116.3 ± 10.6° vs. 113.8 ± 11.4° (*p* = 0.16); and at 12 months, 117.5 ± 10.5° vs. 115.0 ± 11.3° (*p* = 0.15) for CR and PS, respectively. CR knees showed slightly higher flexion at all evaluations, but the differences were not statistically significant.

**Figure 6:**
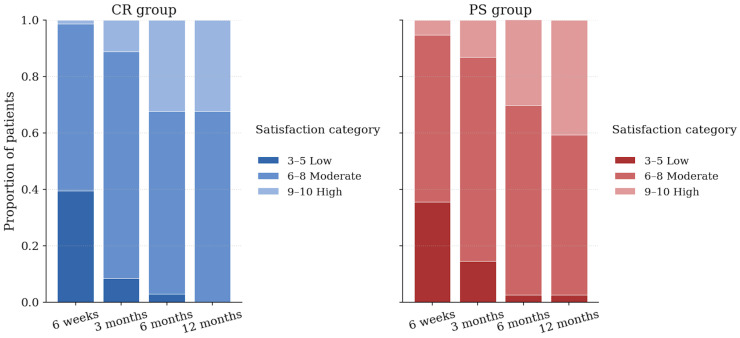
Distribution of patient satisfaction using a 0–10 numerical rating scale over time for cruciate retaining (blue) and posterior stabilized (red) TKA. Values expressed as proportions of patients within each satisfaction category at 6 weeks, 3 months, 6 months, and 12 months postoperatively.

Patient satisfaction increased progressively after surgery in both groups (Figure 6). At 6 weeks, a substantial proportion of patients in both cohorts still reported low or moderate satisfaction levels.

As expected, by 3 months, the majority had reported a moderate satisfaction level. At 6 and 12 months, most patients in both groups reported high satisfaction. The distribution of satisfaction levels was similar between CR and PS implants at each timepoint. Satisfaction scores were high in both groups at 12 months, with CR scoring 8.14 ± 1.02 and PS scoring 8.17 ± 1.22 on the 0–10 numeric rating scale (*p* = 0.87, Cohen’s *d* = 0.03).

A detailed overview of functional outcomes during the follow-up period is presented in Table 4.

Table 5 illustrates PROMS and quality-of-life measurements and detailed statistics for both groups.

Early complications were minor in both groups. Two patients in the PS group experienced delayed wound healing, and one patient in the CR group developed a small area of superficial wound necrosis. All events resolved with local wound care and, when indicated, a short course of oral antibiotics. No deep infections, thromboembolic events, stiffness requiring manipulation under anesthesia, periprosthetic fractures, or early revisions occurred during follow-up.

## 4. Discussion

In this prospective, an Eastern European cohort of younger patients undergoing TKA, assessing both CR and PS implants, demonstrated improvements in pain, function, and quality of life with only small differences between groups. The results align with growing data indicating no major outcome differences between CR and PS TKA designs in outcome measures. Although some outcomes showed small numerical differences between CR and PS implants, none approached thresholds for minimal clinically important differences with limited clinical relevance at 12 months. Given that our sample size exceeded the calculated power requirement to detect an MCID of 9 points, and the observed difference was far below, we can confidently state that no clinically meaningful difference exists between the groups in this cohort. Younger and highly active patients are theoretically more sensitive to subtle kinematic advantages, yet our data did not reveal any consistent trend towards one design. The similarity in PROMs, ROM, functional tests, and satisfaction indicates that these small numerical differences are unlikely to represent a meaningful advantage in early postoperative recovery.

Implant selection was based on PCL integrity and not on radiographic grade alone, and we acknowledge that severe OA can be associated with PCL degeneration. Although intraoperative PCL assessment is the most accurate method of deciding between CR and PS, it can still introduce subtle selection bias because only knees with preserved PCL integrity, stable, and with optimal soft-tissue conditions are eligible for CR. However, OA grading was comparable between groups. It is also worth noting that all TKAs in this cohort were performed using contemporary modern-generation CR and PS components (Smith & Nephew, Legion Genesis II CR/PS systems). A 10-year follow-up study of contemporary CR vs. PS TKAs showed equivalent functional scores, range of motion, patient satisfaction, and implant survival, concluding that neither design proved superior and both provided durable, successful outcomes [16]. These consistent findings in heterogeneous populations endorse the observations for our younger cohort: CR and PS implants both alleviate KOA symptoms and improve knee function in the medium term. One commonly cited distinction is in maximal flexion and kinematics. The PS design cam-and-post mechanism is intended to substitute for the PCL, facilitating femoral rollback at high flexion angles. Certain studies report that PS TKAs achieve a few degrees greater maximum flexion than CR TKAs [4,17]. In the present cohort, range of motion improved in both groups and although we did not find a large flexion difference at one year, the literature suggests PS implants can confer a slight flexion advantage on the order of 3–5° [17]. In a recent randomized trial, Tille et al. observed median flexion of 120° in PS knees vs. 115° in CR knees at 2 years (*p* = 0.017), confirming a small but significant flexion benefit with PS [4]. Despite better flexion in PS knees, overall PROMs and subjective satisfaction were statistically indistinguishable between CR and PS cohorts. Our finding of a non-sustained improvement at 3 months in PS group may indicate a faster early recovery in flexion or function with PS. However, it is important to note that this flexion benefit does not clearly translate into superior patient reported function or satisfaction. Similarly, our patients reported equivalent improvements in pain relief, daily function, and quality of life irrespective of implant type. This suggests that a few degrees of motion difference may have little impact on typical activities for most patients within the immediate period post-surgery.

Another type of outcome evaluated is patient satisfaction and joint perception. Our study noted high satisfaction in both groups, with no significant between-group difference. Serna-Berna et al. reported no difference in patient satisfaction between CR and PS even at 10 years follow-up, with both groups exceeding 90% satisfaction. However, their cohort underwent TKA at a mean age of around 70 years, compared with 58 years in the present cohort. Older patients generally report higher satisfaction and better pain and quality-of-life outcomes after TKA than younger patients, although satisfaction is multifactorial and influenced by factors such as mental health, disease severity, and preoperative expectations [18,19].

When TKAs are well balanced and pain relief is achieved, the specific implant philosophy is not a primary driver of whether patients are satisfied or not. Our inclusion of a mental health outcome (PROMIS Depression) offers a new perspective, as few comparative studies have examined psychological metrics by implant type [20]. Both groups in our cohort saw improvements in PROMIS Depression scores post-TKA but there was no meaningful difference between CR and PS in psychological outcomes. This is in accordance with expectations that the mental health improvement rises from an overall successful surgery rather than specific implant design [21]. A recent study included PROMIS Global Health scores and reported a slightly higher 2-year mental health T-score in CR patients than PS [20]. The authors attributed this to better pain relief and function in their CR group, which may have positively impacted emotional well-being. In our study, since pain and function outcomes were equivalent between designs, it follows that mental health improvements were also equivalent.

Given the largely similar outcomes, it is worth exploring why some studies have reported divergent findings or small advantages for one design. Younger TKA patients tend to report slightly lower satisfaction and more residual symptoms than older patients, despite equivalent objective improvements [22]. Previous studies in working-age cohorts report high overall return-to-work rates but also show that a relevant proportion of patients remain dissatisfied with knee-straining work tasks, despite good pain and function scores [6]. This has been attributed to higher functional aspirations of younger individuals that are critical of any limitations in knee function (difficulty with high-impact activities or full flexion) and thus less likely to report the result as “perfect” [23]. In our study, even though satisfaction was high in both groups, we confirm that any subtle design-related differences (such as PS potentially allowing deeper knee flexion) might be more noticeable or relevant to a younger demographic.

Some authors suggest that surgeon experience and familiarity with a given design outweigh the design differences themselves in determining outcomes [4]. In our cohort, the procedures were performed by the same two senior surgeons with equal experience in CR and PS types over the past 20 years. Both surgeons followed the same predefined intraoperative selection algorithm which reduced preference based variation.

The slight flexion advantage with PS is mechanistically plausible as the cam mechanism provides posterior femoral translation at mid-to-high flexion maintaining femoral rollback that might otherwise be lost if the PCL is removed [17]. A CR knee relies on the preserved PCL for rollback; if the PCL is tight, it could limit flexion and sometimes manifests as “paradoxical anterior femoral slide” [17]. This might explain why some studies on older-generation implants or suboptimal PCL balancing found inferior flexion in CR designs [24]. However, modern CR implants combined with optimal surgical technique can achieve excellent flexion in most patients and gait analyses have shown no significant difference in overall knee motion during walking between CR and PS designs. In fact, Li et al., in an analysis of gait parameters, concluded that aside from maximal bending angle, kinematic patterns, walking speed, and Knee Society Score outcomes were equivalent between CR and PS TKA during level walking [17]. PS implants also carry the risk of patellar “clunk” or clicking due to fibrous tissue catching on the femoral cam, whereas CR implants can sometimes lead to PCL-related issues such as patellar sag or tightness, a potential cause of anterior knee pain [15]. The incidence of these complications is low, but they have been reported. In our series, there were no differences in complication rates. Outcome differences between CR and PS TKAs are minimal in the modern era. Our results suggest that in appropriately selected younger, active patients, surgeons can confidently use either CR or PS designs, with implant choice individualized according to PCL integrity, limb alignment, bone loss, and functional goals rather than implant philosophy alone.

### Limitations

Follow-up was limited to 12 months with interim 3-month data, which capture early outcomes but not longer-term differences. Many of the between-group differences we noted were transient and had plateaued by one year; however, subtle diverging trends (in either clinical scores or wear related issues) could emerge beyond the first postoperative year. Ongoing follow-up of this cohort will be important to determine if the small early advantages of PS are sustained or nullified over time, and whether any differences in failure rates or late complications manifest. The study was prospective but lacked randomization. While this introduces a risk of selection bias, implant selection was driven by a strict anatomical algorithm (PCL integrity) and not surgeon preference. This reflects real world clinical practice, whereas randomizing a PCL-deficient knee to a CR implant would be unethical. The operating surgeons were not blinded to implant type, which can introduce some performance bias, although this was mitigated by the use of a predefined selection algorithm, two senior surgeons with comparable experience in both designs, and a standardized rehabilitation protocol. Implant type was determined by surgeon discretion and intraoperative assessment of each patient PCL and knee stability. We attempted to minimize selection bias by enrolling a consecutive series with similar baseline characteristics, but unknown variables could influence outcomes. The literature shows that when allocation is randomized, outcomes between CR and PS are consistently similar [16].

This cohort exclusively involved patients under 65 years of age. Older patients might have different absolute outcome scores or implant preferences, although prior studies suggest the relative comparison of CR vs. PS holds true [14]. This analysis evaluated standard PROMs and a depression score but did not specifically measure joint awareness or activity levels beyond general health. We also did not record return-to-work status or use activity-focused PROMs or performance-based tests, which would be valuable outcomes in this younger predominantly working-age cohort. Future studies could incorporate those to see if younger patients discern any experiential differences between CR and PS designs in daily activities involving the knee.

## 5. Conclusions

Younger, active patients can expect excellent outcomes from TKA with either CR or PS implant types. Both CR and PS TKA designs achieved statistically indistinguishable outcomes in younger patients with significant pain relief, high satisfaction, functional recovery, and improvements in mental health at one year after the surgery. The modest early advantages observed with the PS design in isolated scores did not translate into sustained clinical superiority. Surgeons should base the CR vs. PS decision on individual patient anatomy and ligament status, as well as on their own experience, rather than on a perceived large outcome difference. Proper surgical technique and patient expectations will likely have a far greater impact on post-TKA success than the choice of retaining or substituting the PCL. These findings contribute to the evidence that CR and PS TKAs are more alike than different in terms of patient-centered outcomes.

## Figures and Tables

**Table 1:** Baseline demographic and clinical characteristics of the study population.

Characteristic	PS (*n* = 76)	CR (*n* = 71)
Female, yes (%)	41 (53.9)	43 (60.6)
Age, years, mean ± SD	57.2 ± 6.1	58.7 ± 6.4
BMI, kg/m^2^, mean ± SD	29.1 ± 4.2	28.7 ± 4.4
Rural residence, no (%)	32 (42.1)	28 (39.4)
Smoker, yes (%)	18 (23.7)	15 (21.1)
Alcohol use, regular, yes (%)	12 (15.8)	11 (15.5)
Employed at time of surgery, no (%)	21 (27.6)	20 (28.2)

**Table 2:** Distribution of Ahlbäck grades between CR and PS groups.

Ahlbäck Grade	PS	CR	*p*-Value
Grade III, no (%)	18 (23.7)	20 (28.2)	0.72
Grade IV, no (%)	28 (36.8)	32 (45.1)	0.41
Grade V, no (%)	30 (39.5)	19 (26.8)	0.09

**Table 3:** Between-group differences (PS vs. CR) in ΔLEFS and MCID.

Follow-Up	Mean ΔLEFS *	95% CI Low	95% CI High	*p*-Value	MCID (%)	95% CI Low	95% CI High
6 weeks	0.57	−0.14	1.27	0.113	17.1	2.1	31.1
3 months	0.27	−0.94	1.48	0.661	5.9	−4.9	15.6
6 months	−0.28	−1.88	1.31	0.724	6.0	−5.3	16.0
12 months	0.14	−1.61	1.88	0.876	6.3	−5.8	16.3

* Mean ΔLEFS is the subject change from baseline between CR and PS. Proportion CIs use the Wilson method; differences in proportions use Newcombe–Wilson intervals. Between-group differences in mean change use Welch’s *t*-test with 95% CIs.

**Table 4:** Summary of mean values, standard deviations, and between-group *p*-values for functional outcomes following total knee arthroplasty (CR vs. PS).

	Baseline			6 Weeks			3 Months			6 Months			12 Months		
	Mean ± SD		*p*	Mean ± SD		*p*	Mean ± SD		*p*	Mean ± SD		*p*	Mean ± SD		*p*
	CR	PS		CR	PS		CR	PS		CR	PS		CR	PS	
LEFS	48.2 ± 14.8	47.0 ± 14.6	0.63	56.2 ± 14.8	55.6 ± 14.6	0.80	65.0 ± 12.8	64.1 ± 12.9	0.66	66.5 ± 11.8	65.0 ± 12.4	0.46	66.1 ± 11.6	65.0 ± 12.43	0.60
Lysholm	56.6 ± 10.0	55.4 ± 10.4	0.47	63.5 ± 10.0	65.6 ± 9.1	0.18	71.2 ± 11.4	69.3 ± 10.8	0.30	75.6 ± 11.2	76.3 ± 10.7	0.69	89.0 ± 11.4	86.7 ± 10.6	0.20
Flexion	94.6 ± 9.9	93.0 ± 10.5	0.34	99.7 ± 10.5	97.9 ± 11.5	0.32	111.7 ± 10.7	109.3 ± 11.7	0.20	116.3 ± 10.6	113.8 ± 11.4	0.16	117.5 ± 10.5	115.0 ± 11.3	0.15

**Table 5:** Mean values, standard deviations, and between-group *p*-values for patient-reported outcome measures and quality-of-life outcomes following TKA.

	Baseline			6 Weeks			3 Months			6 Months			12 Months		
	Mean ± SD		*p*	Mean ± SD		*p*	Mean ± SD		*p*	Mean ± SD		*p*	Mean ± SD		*p*
	CR	PS		CR	PS		CR	PS		CR	PS		CR	PS	
EQ-5D-5L	0.57 ± 0.13	0.54 ± 0.14	0.12	0.64 ± 0.15	0.60 ± 0.15	0.11	0.73 ± 0.16	0.68 ± 0.16	0.95	0.75 ± 0.16	0.70 ± 0.16	0.87	0.76 ± 0.16	0.72 ± 0.16	0.07
VAS	7.39 ± 1.19	7.55 ± 1.46	0.47	3.77 ± 1.21	4.07 ± 1.57	0.20	2.45 ± 1.09	2.82 ± 1.27	0.06	1.86 ± 0.95	1.87 ± 1.08	0.95	1.59 ± 0.84	1.75 ± 0.90	0.27
PROMIS	56.06 ± 7.7	54.95 ± 8.6	0.41	51.82 ± 6.5	53.11 ± 7.1	0.25	51.63 ± 6.5	52.20 ± 7.5	0.62	50.23 ± 6.8	51.53 ± 6.2	0.23	47.45 ± 6.6	48.29 ± 6.5	0.44
Satisfaction	n/a	n/a	n/a	5.83 ± 1.40	6.01 ± 1.53	0.45	7.23 ± 1.24	7.04 ± 1.37	0.39	7.97 ± 1.13	7.92 ± 1.19	0.79	8.14 ± 1.02	8.17 ± 1.22	0.87

## Data Availability

The data are not publicly available due to privacy or ethical restrictions and are available upon request from the correspondent author.

## Abbreviations

The following abbreviations are used in this manuscript:

KOA	Knee osteoarthritis
TKA	Total knee arthroplasty
PS	Posterior-stabilized (implant/design)
CR	Cruciate-retaining (implant/design)
PCL	Posterior cruciate ligament
OA	Osteoarthritis
PROMs	Patient-reported outcome measures
LEFS	Lower-Extremity Functional Scale
EQ-5D-5L	EuroQol five-dimension, five-level index
VAS	Visual analogue scale
PROMIS	Patient-Reported Outcomes Measurement Information System Depression
FJS	Forgotten Joint Score
BMI	Body mass index
SD	Standard deviation
CI	Confidence interval
POLO	Pull-Out–Lift-Off test
